# A multi-centre survey on hospital malnutrition: result of PNSI study

**DOI:** 10.1186/s12937-021-00741-1

**Published:** 2021-10-28

**Authors:** Somayeh Poudineh, Forough Shayesteh, Jamshid Kermanchi, Ali-Akbar Haghdoost, Parisa Torabi, Yahya Pasdar, Mohsen Azimi-Nezhad, Mohammad Safarian, Majid Hajifaraji, Saeed Eslami-Hasan-Abadi, Omid Pournik, Bahareh Barkhidarian, Abdolreza Norouzy

**Affiliations:** 1 Department of Nutrition, Faculty of Medicine, Mashhad University of Medical Sciences, P.O. Box: 91779-48464, Pardise Daneshghah, Azadi Square, Mashhad, Iran; 2 Ministry of Medical and Health Education, Tehran, Iran; 3 Department of Biostatics and Epidemiology, Public Health School, Kerman University of Medical Sciences, Kerman, Iran; 4 Department of Nutritional Sciences, Health Research Center, Kermanshah University of Medical Sciences, Kermanshah, Iran; 5 Department of Basic Medical Sciences, Neyshabur University of Medical Sciences, Neyshabur, Iran; 6 National Nutrition and Food Technology Research Institute and Faculty of Nutrition Sciences and Food Technology, Shahid Beheshti University of Medical Sciences, Tehran, Iran; 7 Department of Medical Informatics, Mashhad University of Medical Sciences, Mashhad, Iran; 8 Department of Community Medicine, School of Medicine, Iran University of Medical Sciences, Tehran, Iran

**Keywords:** Prevalence, Malnutrition, Subjective global assessment

## Abstract

**Background:**

Disease-related malnutrition is associated with adverse outcomes such as increased rates of morbidity and mortality, prolonged hospital stay, and extra costs of health care. This study was conducted to assess nutritional status among patients and to determine the risk factors for malnutrition in Iran university f.

**Methods:**

Persian Nutritional Survey In Hospitals (PNSI) was a cross-sectional study that conducted in 20 university hospitals across Iran. All the patients with age range of 18 to 65 years, who were admitted or discharged, were assessed by subjective global assessment (SGA).

**Results:**

In total, 2109 patients were evaluated for malnutrition. Mean values of age and body mass index were 44.68 ± 14.65 years and 25.44 ± 6.25 kg/m^2^, respectively. Malnutrition (SGA-B & C) was identified in 23.92% of the patients, 26.23 and 21% of whom were among the admitted and discharged patients, respectively. The highest prevalence of malnutrition was in burns (77.70%) and heart surgery (57.84%) patients. Multivariate analysis presented male gender (OR = 1.02, *P* < 0.00), malignant disease (OR = 1.40, *P* < 0.00), length of hospital stay (OR = 1.20, *P* < 0.00), and polypharmacy (OR = 1.06, *P* < 0.00) as independent risk factors for malnutrition. Malnutrition was not associated with age (*P* = 0.10).

**Conclusion:**

This study provides an overall and comprehensive illustration of hospital malnutrition in Iran university hospitals, finding that one out of four patients were malnourished; thus, appropriate consideration and measures should be taken to this issue.

**Supplementary Information:**

The online version contains supplementary material available at 10.1186/s12937-021-00741-1.

## Background

Disease-related malnutrition is associated with adverse outcomes such as increased rates of morbidity and mortality, prolonged hospital stay, impaired wound healing, high readmission rate, and increased costs for health care [1–4].

Malnutrition development is associated with insufficient dietary intake or malabsorption, increased nutritional needs, complications of the underlying disease, or a combination of these factors [5]. There is evidence that showed other factors are associated with malnutrition and affect its prevalence such as higher age [6, 7], weight loss [6], polypharmacy [8], educational level, health care system, and economic situation of the country where the study was performed [6, 9].

Studies performed in the United States indicate that approximately 32.7% of hospital patients are either malnourished or at nutritional risk [10], they also indicated that 11–45% of patients in England hospitals and home care suffer from malnutrition [9].

There are different reports of malnutrition prevalence in Iran, Hosseini reported 5.7% of patients on admission and 11% of discharged patients had malnutrition based on body mass index (BMI) ≤18.5 kg/m^2^ [11]. The prevalence of malnutrition among the cancer patients was reported as 53.1%, out of which 29.1% had moderate and 24% had severe malnutrition based on PG-SGA [12]. Forty-three percent of hemodialysis patients were moderately malnourished by SGA [13]. Another study reported that 23% of admitted patients had mild to moderate malnutrition and 6% had severe malnutrition based on SGA [14]. A recent study reported malnutrition rate of 32.62% by Nutrition Risk in Critically ill (NUTRIC) score (included of 1321) in ICU patients of Iran hospitals [15].

Proper nutritional status can play a notably important role in lowering the incidence of the malnutrition-disease defective cycle. Despite the importance of disease-related malnutrition and the great economic costs of this condition imposes on health systems, there is no comprehensive data on disease-related malnutrition and related risk factors in Iran hospitals. Therefore, this study aimed to assess nutritional status among patients in Iran hospitals and to determine different risk factors for malnutrition in hospitalized patients.

## Methods

Persian Nutritional Survey In Hospitals (PNSI) is a multicenter, cross-sectional study conducted in 20 public hospitals which were selected based on a random stratified-cluster method. The sample size was estimated as 2100 patients, based on the relevant formula [16], and data of the study by Norouzy et al. (*P* = 0.32, Z = 95%, d = 0.02) [17]. Informed consent was obtained from all the patients recruited in this study. This study was approved by the Ethics Committee of Mashhad University of Medical Sciences (number 920923). Two educational sessions were held for investigators and a written instruction about data collection was provided.

In each hospital, the investigators referred to included clinical wards and assessed newly admitted or discharged patients on a specified date consecutively. The inclusion criteria comprised of admitted or discharged patients within the age of 18 to 65 years with Iranian nationality, also we excluded duplicated patients. Patients undergoing surgery on the data collection day, outpatients, patients with trauma or eating disorders, and patients admitted to maternity, obstetric, pediatric, orthopedic, and emergency departments or intensive care units (ICUs) were excluded from the study. The study was conducted from 24th to 28th November 2015. STROBE checklist of study is available as supplementary material 1.

### Data collection

Patient characteristics, i.e., gender, date of birth (age), underlying disease, the main affected organ, comorbidity, the number of different prescriptions per day, and history of ICU stay and surgery were recorded. The length of hospital stay was calculated for discharged patients from the date of admission and date of the survey. Body weight was measured by standard Seca scale (Seca 620, Germany) in light clothes to the nearest 1 kg. Body height was assessed by Seca portable stadiometer (Seca 213, Germany) to the nearest 1 cm. Mid-arm circumference (MAC) was measured in mid-acromion and olecranon process interval at the non-dominant relaxed arm with a non-stretchable tape measure to the nearest 0.1 cm.

### Nutritional status

The nutritional status of patients was assessed by subjective global assessment (SGA) and anthropometric measures (BMI and MAC) for all the admitted and discharged patients included in the study [18].

SGA is a valid and reliable tool for assessing nutritional status in hospitalized patients [19, 20]. Among the recommended screening tools, SGA enjoys the highest diagnostic accuracy for acute care patients. Baker et al. [19] and Detsky et al. [20] demonstrated that the use of SGA for evaluating patients yields reliable results with inter-observer reliability of 80%. SGA is comprised of two components: medical history and physical sign. In the medical history part, the severity and pattern of weight loss, dietary intake, gastrointestinal symptoms, and functional capacity are evaluated. In the physical signs part, loss of subcutaneous fat, muscle mass, and presence of edema and ascites were assessed. According to this tool, patients are classified as well-nourished (SGA-A), moderately malnourished (SGA-B), and severely malnourished (SGA-C) [14].

Body mass index (BMI; weight/height^2^) is most commonly used for assessing nutritional status. BMI was calculated as weight (kg) divided by squared height (m^2^) [21]. If BMI was less than 18.5, between 18.5 and 25, and higher than 25, the patient was considered as malnourished, normal, and overweight or obese, respectively.

### Statistical analysis

Quantitative variables with normal distribution were expressed as mean, standard deviation, and range. Quantitative variables with non-normal distribution were reported as median and range. Stratified variables were reported as frequency and percentage. Analysis of nominal qualitative data was performed by non-parametric tests such as the Chi-squared test. Mann-Whitney test was used for ordinal qualitative variables; for quantitative variables with normal distribution, t-test was run. Odds ratios (OR) were reported with 95% confidence interval. To identify independent risk factors logistic regression was used. A *P*-value less than 0.05 was considered statistically significant.

## Results

### Patient characteristics

**Fig. 1:**
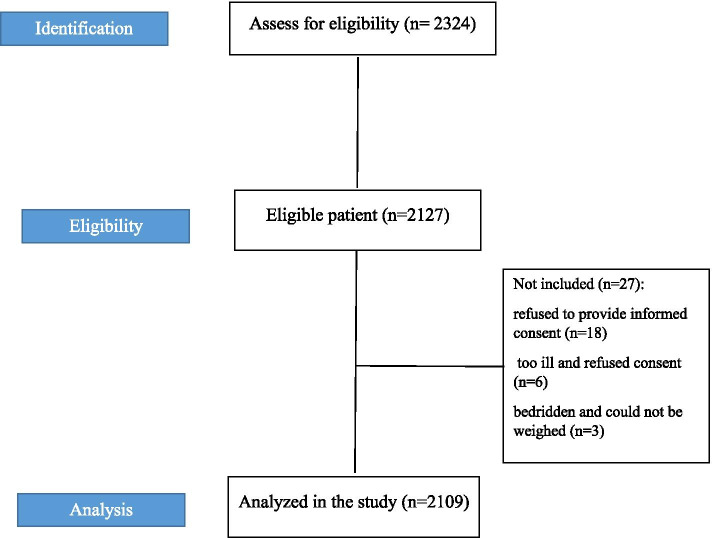
Participant flow chart

Participant flow chart is presented in Fig. 1, 2324 patients were assessed for eligibility, finally data of 2109 patients were analyzed. Patients from 100 medical wards of 20 hospitals were assessed in this study. The mean age of the participants was 44.68 ± 14 years of whom 50% (*n* = 1055) were male and 50% (*n* = 1054) were female. Demographics of the study population are shown in Table 1. Moreover, the mean values for weight, height, and BMI were 68.18 ± 16.53 kg, 164.43 ± 9.75 cm, and 25.44 ± 6.25 kg/m^2^, respectively. In 8.72% (*n* = 183) of the patients, BMI was less than 18.50 kg/m^2^.

**Table 1:** Demographic characteristics of the patients

**Variable**	**Results**			***P*-value**
	**All patients**	**Admitted**	**Discharged**	
**Gender**^**a**^				
Female	1054 (50%)	534 (50%)	494 (50%)	0.76
Male	1055 (50%)	526 (50%)	500 (50%)	
**Age (year)**^**b**^	44.68 (±14/65)	45.43 (±14.96)	43.82 (±14.20)	0.01
**Height (cm)**^**b**^	164.43 (±9.75)	164.1 (±9.66)	164.51 (± 9.77)	0.31
**Weight (kg)**^**b**^	68.18 (±16.53)	67.73 (±16.83)	68.62 (±16.28)	0.24
**Body mass index (W/H**^**2**^**)**^**b**^	25.44 (±6.25)	25.27 (±6.15)	25.60 (±6.21)	0.20
**Mid Arm Circumference (cm)**^**b**^	31.02 (±6.12)	30.03 (±10.23)	32.01 (±9.10)	0.00
**Length of hospital stay**^**c**^	Mean: 6.72 Median: 5 Mode: 3		Mean: 6.72 Median: 5 Mode: 3	
**Kind of drug**^**b**^	3 (±3.21)	2.61 (±3.37)	3.30 (±3.14)	< 0.00
**Number of drugs**^**a**^				
**- No drug**	492 (22.32%)	285 (25.83%)	178 (17.22%)	< 0.00
**- 1 to 2**	617 (27.91%)	293 (26.15%)	307 (29.82%)	
**- 3 to 5**	539 (24.38%)	264 (23.2%)	260 (25.19%)	
**- More than 5**	402 (18.24%)	164 (14.80%)	231 (22.35%)	
**- I don’t know**	56 (7.01%)	96 (8.67%)	54 (5.23%)	
**Mobility**^**a**^				
**Yes**	1770 (82.23%)	825 (78.61%)	877 (86.32%)	
**Only with help**	263 (12.23%)	146 (13.87%)	101 (9.93%)	
**Bed ridden**	90 (4.21%)	63 (6.01%)	25 (2.52%)	
**Comorbidity**^**a**^	1038 (48.72%)	530 (49.31%)	482 (48.33%)	0.64
**- High blood pressure**^**a**^	473 (22.24%)	246 (22.94%)	220 (22%)	0.67
**-Type 1 diabetes**^**a**^	50 (2.28%)	17 (1.62%)	32 (3.25%)	0.01
**- Type 2 diabetes**^**a**^	331 (15.50%)	166 (15.41%)	158 (15.86%)	0.80
**- Chronic respiratory disease**^**a**^	87 (41.23%)	47 (4.43%)	39 (3.98%)	0.59
**- Myocardial infarction**^**a**^	28 (1.32%)	15 (1.39%)	12 (1.22%)	0.69
**- Chronic heart failure**^**a**^	126 (5.91%)	60 (5.57%)	65 (6.48%)	0.37

^a^Categorical variables represented as n (%)

^b^Continuous variables represented as mean ± standard deviation for normally distributed data

^c^Continuous variable presented as mean, median, and mode for not normally distributed data

### Nutritional status

The nutritional data of the patients are presented in Table 2. The overall prevalence of malnutrition was 23.92% (*n* = 479 cases), which is determined by combining the SGA-B (moderate malnutrition, 17.33%) and –C (severe malnutrition, 6.59%) also 76.07% (*n* = 1523) of patients were identified as SGA-A (with normal nutritional status).

**Table 2:** Patients’ nutritional status

**Variable**	**Result (n, %)**
**SGA rating**	
**SGA-A**	1523 (76.07%)
**SGA-B**	347 (17.33%)
**SGA-C**	132 (6.59%)
**Nutritional status**	
**Well-nourished**	1523 (76.13%)
**Malnourished**	479 (23.87%)
**Body mass index (kg/m**^**2**^**)**	
**< 18.5**	181 (8.73%)
**18.5–25**	952 (45.12%)
**25–29.9**	602 (28.47%)
**> 30**	373 (17.70%)
**Mid arm circumference**	
**Normal**	2020 (95.52%)
**Mild malnutrition**	56 (2.63%)
**Moderate malnutrition**	39 (1.81%)
**Severe malnutrition**	0 (0.00%)

Weight loss in the last 3 months was reported by 37.31% (*n* = 799) of the respondents, of whom 217 subjects were well-nourished and 36 subjects were malnourished.

Approximately 3 and 0.50% of the patients had severe to moderate edema and ascites, respectively. A significant statistical association was found between BMI and SGA categories (*P* < 0.05). The highest prevalence of malnutrition was observed in burns department (77.70%), followed by cardiac Surgery (57.84%), and hematology departments (50.00%). The lowest number of patients with malnutrition was in the ophthalmology department (2.51%) (Table 3).

**Table 3:** Prevalence of malnutrition as ward type^a^

**Ward type**	**Malnutrition (n, %)**
**General internal**	131 (36.32%)
**Ophthalmology**	1 (2.51%)
**Otorhinolaryngology**	9 (13.77%)
**Heart**	28 (19.53%)
**Glands**	9 (10.21%)
**Hematology**	12 (50.00%)
**Neurosurgery**	19 (28.35%)
**Heart Surgery**	22 (57.84%)
**General Surgery**	78 (16.42%)
**Vascular surgery**	3 (4.71%)
**Liver and Digestive**	34 (38.25%)
**Renal**	25 (20.6%)
**Nerves**	47 (25.17%)
**Poisoning**	1 (14.19%)
**Infectious**	19 (26.34%)
**Skin**	2 (33.32%)
**Lung**	18 (33.91%)
**Rheumatology**	5 (26.26%)
**Cancer**	5 (35.74)
**Burns**	7 (77.70%)
**Gynecology**	6 (10.32%)

^a^Percentage of malnutrition in each ward calculated based on number of malnourished patients divided on all assessed patients in each ward

Analysis of factors which may have been associated with malnutrition shows male gender (OR = 1.023, 1.015-1.031 *P* < 0.001), malignant disease (OR = 1.409, 1.080-1.830 *P* < 0.001), number of medications (OR = 1.066, 1.030-1.104 *P* < 0.001) and length of hospital stay (OR = 1.206, 1.170-1.304 *P* < 0.001) were independent risk factors for malnutrition. Age and disease type were not associated with malnutrition (*P* > 0.05).

## Discussion

This was the first multicenter study to determine the prevalence of malnutrition in patients of general hospitals in Iran. This study indicated that 23.92% of patients suffered from malnutrition. Independent factors associated with malnutrition were as follows: male gender, malignant disease, polypharmacy, and length of hospital stay.

The results of this study are comparable with reports on the prevalence of malnutrition in Europe and the USA, which shows the importance of identifying malnutrition in hospitals throughout the world. In the United States, Abby C. Sauer found that 33% of patients had malnutrition risk (MST score ≥ 2) [10]. In Europe, malnutrition prevalence was reported as 13% in patients [22].

The results of the present study are in line with those of a national study conducted in Germany using SGA that reported 28% of patients suffered from malnutrition. A study in European hospitals showed that the prevalence of malnutrition ranged between 10 and 50% depending on the studied region [12]. A study using SGA in Korea reported a 22% prevalence of malnutrition for hospitalized patients [23]. Evaluation of nutrition risks in Turkish hospitals demonstrated that 15% of patients were at nutrition risk on admission [22]. The prevalence of malnutrition diagnosed as determined by SGA in Australian hospitals was found to be 30% in patients [18].

There are various reports on the prevalence of malnutrition around the world globe, which might be due to features of the studied samples, the tool used to identify malnutrition, and the type of assessed centers. Moreover, it should be noted that the mean age of patients in this study (44.68 years) was less than other studies (63.93 years) performed in Australia and (52.21 years) Latin America, while age was shown to be an independent risk factor for malnutrition. Additionally, most patients in the present study did not have malignant diseases that could affect the prevalence of malnutrition.

The present study did not demonstrate any association between age and malnutrition that could be pertinent to the low mean age of the samples (44.68 years) [8]. In the study performed in Germany, which divided patients into two groups of aged under 65 and equal to or more than 65 years, malnutrition was not found to be associated with age in the group aged less than 65 years, while in those aged more than 65 years, age was identified as an independent risk factor for malnutrition [8]. In the present study, the prevalence of malnutrition in males was 5.20% higher than in females. This finding is in line with the findings of a study conducted in Argentina [24] and inconsistent with the results of a study by Aliabadi et al., while no difference was observed in the frequency between the two genders in another study [6]. The present findings suggest an association between malnutrition and malignant diseases. Lack of energy protein intake in cancer patients might be due to anorexia and other complications of chemotherapy, e.g., nausea, vomiting, changes in the sense of taste, xerostomia, and early satiety [13, 25]. The multivariate analysis reflected that malnutrition patients at discharge compared to those without malnutrition had a longer duration of hospital stay. Studies using various assessment tools have also shown length of hospitalization as an important risk factor for malnutrition [11, 21]. In this study, the number of administered medications was considered as an independent risk factor for malnutrition. This association was considered in several other studies [26]. The number of medications was closely related to malnutrition, which similar to benign and malignant diseases, however, it can be concluded that polypharmacy itself acts as a major contributing factor to malnutrition, especially given that a large number of drugs are known to decrease appetite or cause vomiting. Recent evidence confirms a synergistic negative effect of polypharmacy and malnutrition on outcomes of older adults [27].

### Strength and limitation

The current study was the first national survey of hospital malnutrition and associated factors in Iran public hospitals which used a valid and reliable tool for nutritional assessment malnutrition, however, there were some limitations such as didn’t assess of malnutrition trend during hospitalization, because of the cross-sectional design of the study, another limitation was assessment by different investigators in hospitals. In further studies status of nutritional care and management of patients can be assessed, also effect of interventional measures in decline and management of problem could be evaluated.

The data of this study provide an overall and comprehensive illustration of the nutritional status of patients and identify groups at higher risk for malnutrition in Iranian university hospitals. Control and management of this problem need a structural approach that should contain all these aspects: 1- administration of a universal assessment to screening patients for nutritional risk to identify patients with or at risk of malnutrition, 2- handle multidisciplinary supportive nutrition care to provide appropriate nutritional therapy, 3- take measures to promote continuity of care after discharge from acute care hospitals. Notable, improve knowledge and consciousness of clinician’s team to consider malnutrition as a specific disease entity that concludes significant patient and economic outcomes and requires active management.

## Conclusion

This study revealed that almost 24% of patients in public hospitals suffer from malnutrition. Male gender, malignancy, polypharmacy, and length of hospital stay were identified as independent risk factors for malnutrition. While the present study cannot determine to what extent and how malnutrition affects the outcomes, there is evidence showing effects on outcome and health costs, in this regard screening and nutrition interventions can improve the results.

## Supplementary Information

**Additional file 1.** STROBE checklist for nutritional epidemiologic studies.

## Data Availability

All data generated or analyzed during this study are included in this published article. There is no public access to the databases.

## Abbreviations

ICU: Intensive care units
ICU: Mid-arm circumference
BMI: Body mass index
SGA: Subjective global assessment
